# Parents’ Reports of Children’s Physical and Sedentary Behavior Engagement among Parents in Weight Management

**DOI:** 10.3390/ijerph19073773

**Published:** 2022-03-22

**Authors:** Catherine Van Fossen, Haley Kiser, Callie Lambert Brown, Joseph Skelton, Keeley Jean Pratt

**Affiliations:** 1 Department of Psychiatry & Human Behavior, Thomas Jefferson University, Philadelphia, PA 19207, USA; 2 Department of Human Sciences, The Ohio State University, Columbus, OH 43210, USA; kiser.105@osu.edu (H.K.); pratt.192@osu.edu (K.J.P.); 3 Department of Pediatrics, School of Medicine, Wake Forest University, Winston-Salem, NC 27101, USA; calbrown@wakehealth.edu (C.L.B.); jskelton@wakehealth.edu (J.S.); 4 Department of Epidemiology and Prevention, School of Medicine, Wake Forest University, Winston-Salem, NC 27101, USA; 5 Department of Surgery, College of Medicine, The Ohio State University; Columbus, OH 43210, USA

**Keywords:** physical activity, family support, health behavior, weight management, obesity

## Abstract

Background: The purpose of this study was to explore the associations between demographics, family exercise participation, family discouragement of exercise, and the children’s physical and sedentary behaviors to identify specific areas of physical activity intervention for children with parents engaged in medical weight management (MWM). Methods: Parents (*n* = 294) of children aged 2–18 years old were recruited from two university MWM programs to complete a one-time survey. Bivariate analyses tested associations. Results: Parents reported that sedentary activity was higher for children who identified as racial minorities (*t*(141) = −2.05, *p* < 0.05). Mobile phone and tablet use was higher for adolescents compared to school age and young children (*H*(2) = 10.96, *p* < 01) Exercise game use was higher for racial minority children compared to white children (U = 9440.5, z = 2.47, *p* ≤ 0.03). Male children (*t*(284) = 1.83, *p* < 0.07), children perceived to have a healthy weight status (*t*(120) = 4.68, *p* < 0.00), and younger children (*t*(289) = 1.79, *p* < 0.08) all engaged in more strenuous physical activity. Family exercise participation (*t*(162) = −2.93, *p* < 0.01) and family discouragement of exercise (U = 7813.50, z = −2.06, *p* ≤ 0.04) were significantly higher for children in racial minority families. Conclusions: Future work should determine methods to engage children and their parents participating in MWM in physical activities together to ensure that the changes the parents are making with MWM are sustainable.

## 1. Introduction

In 2017–2018, approximately 42% of adults and 19% of children (aged 2–19) had an obese weight status [1,2]. Parental and childhood obesity are highly correlated, with parental obesity identified as one of the strongest predictors for children to develop obesity [3]. Other risk factors for childhood obesity include poor diet [4], limited physical activity [5], and increased sedentary behavior [6]. Specifically, children who are not meeting current daily recommendations for physical activity further increase their risk for obesity [7,8]. Older children are at greater risk, as children’s rates of physical activity decrease with each increase in academic grade [9]. For instance, only 33% of adolescents receive 60 minutes of physical activity per day [8]. Interventions that target physical activity at the family level may support both the parents’ and children’s engagement in higher rates of physical activity and less sedentary behavior.

Family systems theory has been utilized as a theoretical framework to explore family dynamics in weight management contexts with children, parents, and romantic partners, with particular attention on how these dynamics may facilitate or prove to be a barrier for the adoption of health behaviors [10,11,12]. Family systems theory describes how families function as a system of interconnected elements that must be viewed as a whole [13]. This is particularly relevant in the context of adult weight management, where although intervention efforts are tailored to the adult, any children living with the parent are likely to be exposed to these changes. In addition to structural changes (i.e., introduction of fitness equipment into the household), parents may increase their communication with their children about physical activity or diet as they engage in weight management [14]. 

Family-based weight management is the standard of care in pediatrics, where the parents’ and children’s dietary and physical activity behaviors are targeted equally [15,16,17,18]. However, few studies have focused on how parental engagement in adult medical weight management (MWM) programs affects dependent children living in the home, and specifically, how family participation in exercise associates with the children’s own physical activity engagement. Notably, Song and colleagues (2018) assessed parental participation in a commercial weight loss program and its influence on child outcomes [19]. While the study reported a significant correlation between the parents’ and children’s decrease in saturated fat consumption and a decline in family meals eaten outside of the home, they did not find any significant changes in the parents’ and children’s physical activity or an association between their rates of physical activity. 

Other researchers have focused exclusively on parental weight loss surgery and its effects on their children’s physical activity. For example, Watowicz and colleagues (2013) found that parents who underwent weight loss surgery reported that their children had less frequent daily physical activity compared to the children of parents with obesity and no history of surgery [20]. However, Woodard (2011) found the children in their study increased their daily physical activity from pre- to post-parental weight loss surgery [21]. Finally, Pratt and colleagues (2018) reported that parents engaged in weight loss surgery who perceived their child to be overweight/obese reported less family exercise participation compared to parents who perceived their child to have a healthy weight status [22]. 

Given the lack of research published about the effects of parental participation in MWM on children, including family participation in exercise and the children’s physical and sedentary activities, the purpose of this study was to explore associations between demographics, family exercise participation, family discouragement of exercise, and children’s physical and sedentary behaviors to identify future areas of research and interventions for children whose parents are engaged in MWM. Additionally, the historical period marked by the COVID-19 pandemic has heightened the need to better understand how families are engaging in sedentary and physical activity behaviors, as children have not recently experienced a similar time with reduced access to public fitness facilities and school physical education. This study has a descriptive exploratory design based on previous literature regarding family dynamics and the support for family-based prevention and intervention for childhood obesity with the intent of informing future physical activity intervention for the children of parents engaging in weight management. We expected the study variables (sedentary behaviors, mobile device use, exercise game use, physical activity) to differ across demographic categories in alignment with the previous literature indicating that children who are racial minorities, male, overweight/obese, and older will be at a higher risk for physical inactivity and/or sedentary behaviors [5,6,7,8,9,23,24,25]. We also anticipated that family participation in exercise would be positively associated with physical activity and negatively associated with sedentary activity; andfamily discouragement of exercise would be positively associated with sedentary activities and negatively associated with physical activity. 

## 2. Materials and Methods

### 2.1. Procedures

Following Smart IRB approval (Smart IRB #2017B0210), participant recruitment took place from May to November 2017 at the outpatient weight management clinics of two large university medical sites. The targeted sample size was 300 (150 parents from each site). The analytic sample size for this study was 294 participants. Following consent, the parents completed a one-time survey packet prior to exiting the clinic. The survey packet included a variety of measures assessing clinical demographics, weight status, couple and family support, and both physical and sedentary behaviors of the child and the family. For the purpose of this study, we used the parent’s perception of child weight status, family exercise participation, family discouragement of exercise, and the child’s physical and sedentary activities. Further descriptions of this study sample and the above measures have been published elsewhere [12,22]. 

### 2.2. Participants

Recruitment of participants at both sites included various adult outpatient weight management options, including combinations of weight-loss medication management, weight loss surgery, and educational/behavioral weight management programs. Criteria for participation included: English speaking and reading; participant must be ≥18 years old; live in the home for the majority of the week (≥4 days per week) with their child (ages 2–18 years old) and a romantic partner (≥18 years old); must have no known medical conditions or terminal illnesses that would prevent them from participating in their weight management program. If participants had more than one child living in the home that met our inclusion criteria, they were instructed to select the youngest child within the age range when completing the survey. Participants with a child of any weight status were allowed to participate. Participants received a $10 retail gift card for their time in the study. Participants will be referred herein as parents [12,22]. 

### 2.3. Measures

Demographics. Parents provided their age, gender, height (feet, inches), weight (pounds), educational attainment, ethnicity, race, and number of children living in the household. Parents also provided information on their youngest child’s age, gender, weight, height, perceived weight status (underweight, healthy weight, overweight, obese), ethnicity, and race. Perceived weight status was utilized in this study due to the underestimation of height and weight used to calculate weight status among parents with obesity [26,27], and that the perception of weight status is likely more accurate than having parents self-report their child’s height and weight (since the child was not available for measurement). Parents had access to a wall mounted stadiometer and research grade scale in both clinical settings where recruitment was conducted. 

Children’s Sedentary Behaviors. The parents’ perceptions of the amount of time that their children spent doing sedentary activities on both weekdays and weekends were assessed using validated Project EAT questions [28]. Sedentary activities included three items: TV/DVD/videos, using a computer (not for work or school), playing Xbox/Play-Station/or other electronic games while sitting. Each sedentary behavior was assessed for the weekdays and weekend with the following Likert options: 0 h, 0.5 h, 1 h, 2 h, 3 h, 4 h, and 5+ hours. Weekday responses were multiplied by 5 and the weekend by 2 to weight them appropriately for a 7-day week. Weekday and weekend weighted responses were summed to generate a score of hours per week that the child engaged in this activity. This scale has good reliability based on prior studies [29,30]. The reliability in the current study was α = 0.78. 

Children’s mobile device and exercise video games use. Additionally, mobile device and exercise game (e.g., Wii Fit, Wii Sport, Dance Dance Revolution) use were assessed using the same protocol as the sedentary behaviors, but they were assessed as separate items from the sedentary behavior total following the Project Eat scoring protocol [28]. This yielded an estimate for the hours per week of mobile device or video game use. The reliability of mobile use in the current study was α = 0.87. The reliability of exercise game use in the current study was α = 0.84.

Children’s Physical Activity. The parents’ perception of the amount of weekly physical activity their child participated in was assessed using a three-item validated scale for strenuous, moderate, and mild physical activity from Project EAT [28]. Likert responses for strenuous, moderate, and mild physical activity included the following: none, less than 0.5 h, 0.5 to 2 h, 2.5 to 4 h, 4.5 to 6 h, and 6+ hours per week. These questions have a strong reliability based on previous studies [28,29,30,31]. The reliability for these items used as a total score in our study was α= 0.73. 

Family Exercise Participation and Discouragement. Family exercise participation and discouragement of exercise were assessed using the Social Support and Exercise Survey [32]. The Social Support and Exercise Survey contains 10 items for assessing family exercise participation, and 3 items for assessing family discouragement of exercise, each with the following Likert scale responses: never, rarely, a few times, often, very often, and does not apply. Responses were summed to create the overall score for both family exercise participation and family discouragement of exercise. Both scales have demonstrated acceptable reliability and validity [32]. We dropped the rewards item from the rewards and punishment scale to aid in the interpretation of this scale as only reflective of punishment/discouragement, and this improved scale reliability. The reliability of family exercise participation in the current study was α= 0.93, and for family discouragement of exercise, it was α = 0.77. These scales have been used in both clinical and non-clinical settings, and in adult weight management programs [32,33,34]. 

### 2.4. Analysis

All statistical analyses were carried out using the Statistical Package for the Social Sciences (IBM SPSS +, Version 27.0, Chicago, IL, USA). Bivariate analyses (correlations, independent samples *t*-test, and ANOVA) were used to test associations between demographics, family exercise participation, family discouragement of exercise, and children’s physical and sedentary behaviors. Statistical significance was set at *p* < 0.05. Missing data were handled using listwise deletion. The study variables were normally distributed with the exception of the discouragement scale and exercise games use, which were analyzed using non-parametric measures, specifically Spearman’s correlation, Mann–Whitney, and Kruskal-Wallis tests. 

## 3. Results

### 3.1. Study Demographics 

The majority of the parents identified as female (*n* = 249, 84.7%) and white (*n* = 215, 74.4%) with an average age of 41, and all had an obese weight status. For the MWM programs, the parents identified as participating in behavioral weight management (*n* = 102, 34.7%), weight loss surgery (*n* = 143, 48.6%), behavioral weight management and surgery (*n* = 12, 4.1%), and individualized plans or medication-only (*n* = 31, 10.6%). The children were equally split by gender, the majority of which were identified as white (*n* = 191, 67.5%), had an average age of 9 years-old, and based on parent-perception, had a healthy weight (*n* = 230, 79.3%) or an overweight/obese (*n* = 60, 20.7%) weight status. Table 1 further details the demographics of the parents and the children. Table 2 and Table 3 show associations of demographics and study variables. 

### 3.2. Associations with Child Sedentary Behaviors

Child sedentary behaviors (computer, TV, and seated video gaming) were significantly associated with child race (*r*(274) = 0.13, *p* < 0.05) and gender (*r*(279) = −0.13, *p* < 0.05). Racial minority children (M = 21.64, SD = 14.34) were more likely to engage in sedentary behaviors than white children (M = 26.19, SD = 18.37; *t*(141.33) = −2.05, *p* < 0.05). Males (M = 25.09, SD = 17.13) were more likely to engage in sedentary behaviors than females (M = 21.06, SD = 14.24; *t*(271.12) = 2.14, *p* < 0.05). This sedentary behavior total score was also significantly and positively associated with child mobile device and tablet use (*r*(229) = 0.44, *p* < 0.01) and exercise video game use (*r_s_*(278) = 0.24, *p* < 0.01). Child mobile device and tablet use was significantly and positively associated with child age (*r_s_*(231) = 0.19, *p* < 0.01). Mobile device use differed significantly on the basis of age grouping (*H*(2) = 10.96, *p* < 01), with adolescents (13–18; Mdn = 14.00) engaging in significantly higher use when compared to young children (Mdn = 4.00), and moderately higher use when compared to school age children (Mdn = 9.00). Child sedentary behaviors were not significantly associated with any other demographic variables, family participation in exercise, or family discouragement of exercise. 

### 3.3. Associations with Child Physical Activity

Child strenuous physical activity was significantly and positively associated with mild (*r*(287) = 0.31, *p* < 0.01) and moderate (*r*(289) = 0.53, *p* < 0.01) physical activity. Additionally, strenuous physical activity was negatively associated with child weight status (*r*(288) = −0.23, *p* < 0.01) and positively associated with mobile device/tablet use (*r*(229) = 0.17, *p* < 0.01). Male children (M = 3.51, SD = 2.97) were moderately more likely to engage in strenuous physical activity than female children (M = 2.90, SD = 2.72; *t*(284) = 1.83, *p* ≤ 0.07. Children with an overweight or obese weight status (M = 1.92, SD = 2.20) were less likely to engage in strenuous physical activity than children with an underweight/healthy weight status (M = 3.53, SD = 2.93; *t*(120.01) = 4.68, *p* < 0.01). Child strenuous (*r*(291) = −0.10, *p* < 0.08), moderate (*r*(290) = −0.11, *p* = 0.05), and mild activity (*r*(288) = −0.15, *p* < 0.01) were negatively associated with child age. There were no significant differences between young, school-age, and adolescent children for strenuous (F(2,287) = 1.48, *p* > 0.10) or moderate activity (F(2,286) = 1.95, *p* > 0.10); however, there were significant differences by age group for the mild activity (F(2,284) = 3.90, *p* < 0.05) of young and adolescent children. Child exercise game use was significantly and positively associated with child race (*r_s_* (276) = 0.15, *p* ≤ 0.01). Child strenuous, moderate, and mild physical activity had no other significant associations with any other demographic variables, family participation in exercise, or family discouragement of exercise. 

### 3.4. Associations with Family Participation and Discouragement of Exercise

Family participation in exercise was only significantly associated with the children’s race (*r*(283) = 0.18, *p* < 0.01). Parents with children who were racial minorities (M = 28.53, SD = 11.23) were more likely to report that the family displayed higher participation in exercise than parents of non-racial minority children (M = 24.49, SD = 9.97; *t*(162.01) = −3.06, *p* < 0.01). Additionally, family participation in exercise was also significantly and positively associated with child video exercise game use (*r_s_* (286) = 0.19, *p* < 0.01). Family participation in exercise was not significantly associated with any other child demographics, sedentary, or physical activity behaviors. The Mann-Whitney test and Spearmen’s correlation were conducted to explore the associations with family discouragement of exercise due to its non-normative distribution. Family discouragement of exercise was significantly associated with the children’s race (*r_s_*(283) = 0.12, *p* ≤ 0.05). Parents with children who were racial minorities (mean rank = 152.57) were more likely to report that the family displayed more discouraging behavior for exercise than parents of non-racial minority children (mean rank = 136.91, U = 7813.50, z = −2.06, *p* ≤ 0.04).

## 4. Discussion

This study reports, for the first time, associations between the demographics, children’s physical and sedentary behaviors, family exercise participation, and family discouragement of exercise among parents engaged in medical weight management (MWM). Parents with obesity who are engaged in MWM are tasked with adopting new dietary and physical activity behaviors, which may affect the children who are living in the home with them. Family members’ participation in exercise may either aid or hinder the parent’s behavioral changes, and ultimately weight loss. The results obtained in this study will help to determine areas for future research and the need for family-based physical activity interventions for MWM. 

In the current study, child sedentary behaviors (i.e., computer, TV, and seated video gaming) were significantly associated with child race and gender. Mobile device use was also significantly associated with child age, where older adolescents engaged in greater use. Additionally, older children were significantly less likely to participate in any type of physical activity. As younger children age into adolescents, they are placed at greater risk of engaging in sedentary activities [23], decreasing their rates of physical activity, which was also noted in the current study. Parents noted that male children had higher rates of both physical and sedentary activities. Historically, male children have been encouraged to participate in higher rates of physical activity, such as organized sports, and have acceptable or normalized high rates of video game use contributing to their sedentary behavior [24]. While there was no difference in the rate of sedentary behavior by child weight status, there were significant differences in their engagement in physical activity. Interestingly, mobile device use was positively associated with strenuous physical activity. Focusing on increasing the physical activity behavior of parents and their children rather than decreasing screen time may be an important distinction, especially for older children, given the availability of modern exercise equipment that incorporates screens. 

Parents of children in the current study who identified as racial/ethnic minorities reported higher family exercise participation, a higher rate of exercise video game use, and a higher rate of family discouraging behaviors for exercise. It may be that racial/ethnic minority parents in MWM engage with their children in activities that are safe and easy to do year-round, like free play and electronic exercise games, as a way to increase their children’s physical activity [8]. Given the high rates of obesity affecting children from racial/ethnic minority backgrounds [25], this finding may indicate that there are other more significant influences, such as socioeconomic status, on a child’s weight status. Additionally, it was interesting that these families indicated higher family participation and higher discouragement, though neither of these variables were directly significantly associated with physical activity for these children. These families may be undermining well intentioned efforts to enact physical activity change; however, future investigation is necessary to better understand how family dynamics unfold in diverse families. 

This study is not without limitations. This was a descriptive exploratory study where parents self-selected MWM. This study design was chosen given the dearth of research on the children of parents in weight management, especially pertaining to family support and discouragement of physical activity in this context. The exploratory nature of this study precludes any inferences about developmental or longitudinal trajectories for the participants; however, this study serves to provide foundational knowledge for this population. As a result, this study utilized correlational analyses that reported on several relationships with weak correlations. This may suggest that there are other demographic correlates for family support and the discouragement of physical activity that should be explored (e.g., family income). Barriers to resources like socioeconomic status may be helpful in exploring how these dynamics may differ across diverse families. Alternatively, some demographics emerged from this study as worthy for further consideration (e.g., identification as a racial minority). Another limitation of this study was the broad age range of the children, 2–18 years old. This presented concerns regarding interpretation of the results within a developmental context. Subgroup analysis by age was included in the study to address this concern; however, it will likely be important to tailor future interventions for the age of the child. Additionally, our study design included a residency requirement, where the children were required to live with their parents for the majority of the week to address some of the concern about parental influence on the children. This was done to ensure that the child was living with the family for whom exercise participation, support, and discouragement were measured. Another way to improve assessment of family dynamics and its relationship with child physical activity is to solicit information on multiple family members. Although child variables were assessed in our study in addition to parent variables, they were assessed from the parent’s perspective, not through dyadic data collection involving the child. Future research should dyadically assess how a child’s physical and sedentary activities changes throughout parental engagement in MWM, and how family exercise participation and discouragement of exercise may determine changes in both the parent’s and child’s physical and sedentary activities. However, monitoring parental perception may still be important, since perceptions do influence a parent’s approaches to parenting activities (e.g., feeding and exercise). 

## 5. Conclusions

Family-based physical activity interventions in a MWM setting may have the potential to aid parents with their own behavioral changes and weight loss, ensure that healthy physical activity habits are established with their children, and potentially enhance family and parent-child relationships by engaging in a fun and active time together. Racial/ethnic minority families, specifically African American families, may stand to benefit from interventions which enhance existing family support for physical activity engagement. Future work should determine methods for engaging children and their parents participating in MWM in physical activities together to ensure that the changes parents are making in MWM are sustainable, and to potentially prevent the onset of obesity in a high-risk group of children.

## Figures and Tables

**Table 1:** Sample Demographics (*n* = 294).

Variables	M (SD), Range Frequency (%)
Child age	9.35 (4.86), 2–18
Child gender	
Male	149 (51.4%)
Female	139 (47.9%)
Other	2 (0.7%)
Child race	
White	191 (67.5%)
African American	61 (20.7%)
Asian	4 (1.4%)
Bi-racial or multi-racial	27 (9.5%)
Child ethnicity (Hispanic)	
Yes	24 (8.2%)
No	266 (91.7%)
Child weight status category	
Underweight/healthy weight	230 (79.3%)
Overweight/obese	60 (20.7%)
Caregiver age	41.26 (7.06), 25–59
Caregiver sex	
Male	45 (15.5%)
Female	249 (84.7%)
Caregiver race	
White	215 (74.4%)
African American	59 (20.4%)
Asian	1 (.3%)
Bi-racial or multi-racial	12 (4.2%)
Other	2 (.7%)
Caregiver BMI	40.14 (10.08)
Educational attainment	
High school and under	32 (10.9%)
Some college/associates	116 (39.6%)
At least a Bachelor’s degree	145 (49.5%)

**Table 2:** Scale Descriptives and Bivariate Correlations for Study Variables.

		*n*	Mean (SD)	Range	Skewness	Kurtosis	1	2	3	4	5	6	7	8	9	10	11	12
					Mean (SE)	Mean (SE)												
1.	Child Race	283	0.33 (0.47)	0–1	0.75 (0.15)	−1.45 (0.29)	1											
2.	Child Gender	288	1.48 (0.50)	1–2	0.07 (0.14)	−2.01 (0.29)	0.01	1										
3.	Child Weight Status	290	0.21 (0.41)	0–1	1.46 (0.14)	0.12 (0.29)	−0.07	−0.01	1									
4.	Child Age	294	0.33 (0.47)	0–1	0.71 (0.14)	−1.51 (0.28)	−0.05	0.06	0.27 **	1								
5.	Sedentary Behaviors-Hours per Week	284	23.08 (16.05)	0–70	0.83 (0.15)	0.14 (0.29)	0.13 *	−0.13 *	−0.06	0.01	1							
6.	Mobile Use-Hours per Week	231	10.97 (7.86)	0–28	0.48 (0.16)	−0.67 (0.32)	0.07	0.03	0.05	0.19 **	0.44 **	1						
7.	Exercise Game Use-Hours per Week	286	0.34 (0.80)	0–4	2.94 (0.14)	8.90 (0.29)	0.15 *	−0.01	−0.05	−0.07	0.24 **	0.13 *	1					
8.	Strenuous Physical Activity-Hours per Week	291	3.16 (2.87)	0–8	0.61 (0.14)	−1.05 (0.29)	0.11	−0.11	−0.23 **	−0.10	0.06	0.17 **	0.10	1				
9.	Moderate Physical Activity-Hours per Week	290	3.24 (2.71)	0–8	0.59 (0.14)	−0.93 (0.29)	0.09	−0.06	−0.10	−0.11	0.05	0.00	0.05	0.53 **	1			
10.	Mild Physical Activity-Hours per Week	288	3.28 (2.76)	0–8	0.64 (0.14)	−0.95 (0.29)	−0.01	−0.01	0.07	−0.15 **	−0.01	−0.05	0.01	0.31 **	0.58 **	1		
11.	Family Participation in Exe-cise	294	25.47 (10.57)	10–50	0.31 (0.14)	−0.87 (0.28)	0.18 **	−0.01	−0.02	0.00	0.04	0.01	0.19 **	0.08	0.08	0.09	1	
12.	Family Discouragement for Exercise	294	2.52 (1.24)	2–10	3.28 (0.14)	12.68 (0.28)	0.12 *	0.01	0.01	−0.08	0.04	−0.07	0.05	0.05	0.09	0.05	0.03	1

* *p* < 0.05, ** *p* < 0.01; Child race: white 0, racial minority = 1. Child gender: male = 1, female = 2. Child weight status: underweight/healthy weight = 0, overweight/obese = 1. Child age: young and school age = 0, adolescent = 1.

**Table 3:** Mean Differences.

	Sedentary Behaviors						Strenuous Physical Activity					
	*n*	M	SD	*t*	*p*	*d* (L,U)	*n*	M	SD	*t*	*p*	*d* (L,U)
Child race												
White	185	21.64	14.34	−2.05	0.04 *	−0.29 (−0.54, −0.03)	189	2.98	2.75	−1.80	0.07	−0.24 (−0.49, 0.01)
Racial minority	89	26.19	18.37				92	3.66	3.06			
Child gender												
Male	142	25.09	17.13	2.14	0.33 *	0.25 (0.02, 0.49)	147	3.51	2.97	1.83	0.07	0.22 (−0.02, 0.45)
Female	137	21.06	14.24				139	2.90	2.72			
Child weight status												
Under/Healthy weight	223	3.37	2.36	1.13	0.26	0.15 (−0.14, 0.44)	228	3.53	2.93	4.68	0.00 *	0.58 (0.29, 0.86)
Overweight/Obese	58	3.04	1.87				60	1.92	2.20			
Child age												
School age and younger (2–12)	192	3.28	2.30	−0.15	0.88	−0.02 (−0.27, 0.23)	194	3.37	2.87	1.79	0.08	0.22 (−0.02, 0.47)
Adolescent (13–18)	92	3.33	2.29				97	2.74	2.83			
	**Family Participation in Exercise**						**Family Discouragement for Exercise**					
	***n***	**M**	**SD**	***t***	***p***	***d*** (L,U) *	***n***	**M**	**SD**	***t***	***p***	***d*** **(L,U)**
Child race												
White	191	24.49	9.97	−3.06	0.00	−0.39 (−0.64, −0.14)	191	2.38	0.96	−2.21	0.03 *	−0.32 (−0.57, −0.70)
Racial minority	92	28.53	11.23				92	2.75	1.48			
Child gender												
Male	149	25.60	10.60	0.253	0.80	0.03 (−0.20, 0.26)	149	2.49	1.11	−0.58	0.56	−0.07 (−0.30, 0.16)
Female	139	25.29	10.63				139	2.58	1.40			
Child weight status												
Under/Healthy weight	230	25.59	10.74	0.26	0.79	0.04 (−0.25, 0.32)	230	2.53	1.24	−0.04	0.97	−0.01 (−0.29, 0.28)
Overweight/Obese	60	25.18	10.24				60	2.53	1.31			
Child age												
School age and younger (2–12)	196	25.50	10.51	0.06	0.95	0.01 (−0.24, 0.25)	196	2.56	1.23	0.70	0.49	0.09 (−0.16, 0.33)
Adolescent (13–18)	98	25.42	10.76				98	2.45	1.28			

*d* (L,U) = Cohen’s d, with lower and upper confidence interval; * = *p* < 0.05.

## Data Availability

The data presented in this study are available on request from the corresponding author. The data are not publicly available due to limitations of confidential data.
